# Facial Landmarks Determination with Different Digital Scanners: An In Vivo Study

**DOI:** 10.3390/jcm15041500

**Published:** 2026-02-14

**Authors:** Rita Vanessa Alves, Helena Francisco, Ana Catarina Pinto, Gonçalo Bártolo Caramês, João Caramês, Duarte Marques

**Affiliations:** 1 Instituto de Implantologia, Avenida Columbano Bordalo Pinheiro, No. 50, 1070-064 Lisbon, Portugal; alvesrita@edu.ulisboa.pt (R.V.A.); helenafrancisco@campus.ul.pt (H.F.); anapinto2@edu.ulisboa.pt (A.C.P.); caramesgoncalo@gmail.com (G.B.C.); carames@edu.ulisboa.pt (J.C.); 2 Faculdade de Medicina Dentária, Universidade de Lisboa, R. Profª. Teresa Ambrósio, 1600-277 Lisbon, Portugal; 3 LIBPhys-FCT UIDB/04559/2020, Faculdade de Medicina Dentária, Universidade de Lisboa, R. Profª. Teresa Ambrósio, 1600-277 Lisbon, Portugal

**Keywords:** dentistry, facial scanner, anthropometry, technology, three-dimensional

## Abstract

**Background**: The development of facial scanning technology has introduced new methods for facial morphology analysis, progressively shifting from conventional methodology such as direct anthropometry and two-dimensional photography toward three-dimensional digital acquisition. These technologies aim to reduce operator subjectivity, enhance measurement reproducibility, and enable comprehensive facial analysis within digital workflows. **Methods**: Thirty adult volunteers were recruited and provided informed consent. In each participant, twenty-five predefined facial landmarks were identified and nineteen linear interlandmark distances were recorded using three methods: direct anthropometric measurement with a digital caliper (Mitutoyo^®^, USA), a low-cost portable facial scanner (Revopoint^®^), and a professional static facial scanner (RAYFace^®^). Manual anthropometry was used as a clinical refence standard. All measurements were performed by a single trained operator. Trueness was defined as the absolute difference between the reference measurements and the mean of digital measurements, while precision was defined as the standard deviation of repeated digital measurements. Results were expressed as mean values and 95% confidence intervals. **Results**: Overall precision was 0.58 (0.53; 0.62) mm for Revopoint^®^ and 0.43 (0.39; 0.47) mm for RAYFace^®^, corresponding to precision percentages of 1.19 (1.06; 1.33) % and 0.88 (0.78; 0.97) %, respectively. Mean trueness values were 2.16 (2.01; 2.31) mm and 1.92 (1.80; 2.05) mm for conventional-Revopoint^®^ and conventional-RAYFace^®^, corresponding to a trueness impact value of 4.30 (3.87; 4.74) % and 4.08 (3.61; 4.55) %, respectively. Statistically significant differences between scanners were observed for specific landmark locations. **Conclusions**: Within the methodological limitations of this in vivo study—including the use of manual anthropometry as a reference standard, a single-operator design and a predominantly female sample—both facial scanners demonstrated reproducible linear measurements within clinically acceptable thresholds for prosthodontic and esthetic planning applications. The professional static scanner showed superior accuracy, suggesting greater suitability for complex cases requiring higher precision.

## 1. Introduction

Digital technologies have evolved exponentially in the field of dental medicine, promoting a shift from conventional methods to virtually based methodologies in daily clinical and laboratory practice [1,2,3]. Combining facial aspects and proportions with dento-gingival parameters forms the basis for planning a new smile design and final rehabilitation. Nowadays, three-dimensional data can integrate extraoral tissues (facial scanners), intraoral tissues (soft and hard tissues—intraoral scanning IOS file), and craniofacial/skeletal hard tissues (CBCT), enabling the acquisition of a 3D virtual patient [4,5].

New virtual tools can enhance the predictability and efficiency of dental diagnosis, planning, and treatment implementation, especially when the results can be digitally simulated [6].

Facial scanners are essential resources for acquiring diagnostic data and can be employed in treatment planning and outcome assessment, while the gold standard for analyzing facial landmarks remains the use of two-dimensional (2D) photographs or manual measurement devices such as a digital caliper [7,8]. Facial scanning creates 3D facial models in wavefront object (OBJ) or polygon (PLY) file formats, capturing actual skin texture and color [9,10]. This allows for more precise landmark determination than standard tessellation language (STL) files, which only convey surface geometry [10].

Anthropometric facial measurements have long served as a standard in clinical assessment, being cost-effective and reliable; however, they are limited by operator dependency, and time demands [7,11,12,13,14]. Additionally, there is no recording of landmark/reference coordinates for future analysis and missing data cannot be retrieved later [12].

Three-dimensional facial scanning overcomes many of these limitations by enabling objective, reproducible, and digitally stored facial measurements. Different scanning technologies are currently available, including laser-based systems, structured light scanners, and stereophotogrammetry, each presenting distinct advantages and limitations in terms of accuracy, acquisition time, sensitivity to environmental conditions, and cost [15,16].

Handheld laser scanners are generally affordable and portable but require longer acquisition times and may be more susceptible to motion artifacts, achieving reported accuracies of approximately 1.0 mm [13,17,18,19,20].

Structured light scanners project a pattern of infrared or visible lights onto a surface using the “triangulation method” and obtain the 3D shape from the distortion of the projected object with higher accuracy, up to 0.3 mm. However, these systems are very sensitive to room/environmental lighting conditions [21,22].

Stereophotogrammetry systems compute a 3D shape from photographs acquired from two or more cameras at different angles, and the algorithm produces the three-dimensional image through the reflection of ambient light. In these stereo-camera systems, different technologies work in a synchronized manner. The main advantages of this type of scanner are a short capture time and the ability to operate efficiently in a wide range of environments. The accuracy of these systems is very high, up to 0.1 mm, making them particularly suitable for clinical applications [23,24].

Facial scanning has demonstrated clinical relevance in prosthodontics, particularly for full-mouth rehabilitation planning, esthetic evaluation, and interdisciplinary communication. The integration of facial scans into digital workflows enhances patient communication, allows the simulation of treatment outcomes, and supports the construction of a comprehensive virtual patient [25,26].

Despite these advantages, scientific evidence regarding the trueness and precision of facial scanners in dental medicine remains limited. Furthermore, the superimposition of facial images acquired at different time points still poses challenges, highlighting the need for further validation studies [15,25,27,28].

Therefore, the aim of this in vivo study was to compare the trueness and precision of facial linear measurements obtained using conventional and digital facial measurements and to evaluate different facial scanning systems using a low-cost portable facial scanner and a professional static scanner. The null hypotheses were that no statistically significant differences would be observed in trueness and precision between conventional and digital measurements, and that no statistically significant differences would exist between the two facial scanners.

## 2. Materials and Methods

### 2.1. Study Design/Patient Selection

An in vivo clinical study was carried out between January and July of 2024 in accordance with The World Medical Association’s Declaration of Helsinki. Ethical approval was obtained from the Ethics Committee of the Implantology Institute (II2019-07) and registered at ClinicalTrials.gov (U. S. National Library of Medicine, Bethesda, MD, USA) with the registration number NCT04050878.

Thirty volunteers over 18 years of age were recruited from a private clinical setting. Exclusion criteria included the presence of facial hair, beard, mustache, craniofacial deformities, ongoing facial or intraoral treatment, and systemic conditions that contraindicate exposure to flashing lights. All participants received detailed information about the study and provided written informed consent.

### 2.2. Patient Preparation and Soft Tissue Landmarks

Patients were instructed to avoid make-up, earrings, glasses, facial products, or accessories during the facial scan procedure. Patients with long hair were asked to have it styled and the use of a ribbon or cap was considered when conditions required.

**Scheme 1:**
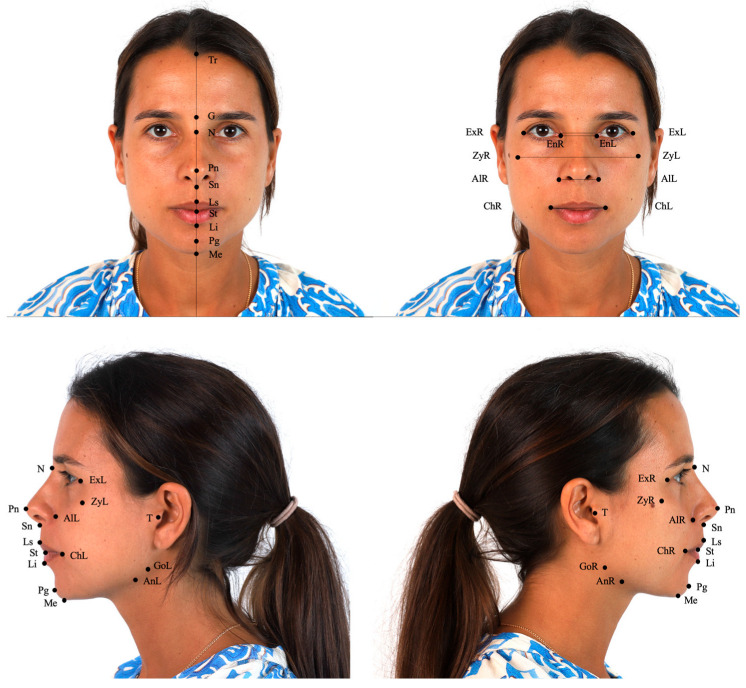
Demographic points marked on patient’s face.

Twenty-five predefined facial landmarks, previously described by Farkas [29], Anas et al. [30], Jayaratne and Zwahlen [31] and Katina et al. [32], were marked on the patient’s face with a demographic marker in the following positions: Trichion (Tr), Glabella (G), Nasion (N), Right Exocanthion (ExR), Left Exocanthion (ExL), Left Endocanthion (EnL), Right Endocanthion (EnR), Right Zygion (ZyR), Left Zygion (ZyL), Right Alare (AlR), Left Alare (AlL), Pronasale (Pn), Subnasal (Sn), Labrale superius (Ls), Stomion (St), Labrale inferius (Li), Pogonion (Pg), Menton (Me), Tragion (T), Right Gonion (GoR), Left Gonion (GoL), Right Antegonion (AnR), Left Antegonion (AnL), explained in Scheme 1 and described in Table 1.

Nineteen linear distances were measured between selected landmark pairs across frontal, horizontal, and sagittal planes, as detailed in Table 2.

The data were collected by one independent operator (R.V.A.) using three different measurement methods: clinically on the patient’s face with a digital caliper (manual group), digitally on a 3D facial scan (Revopoint^®^, Model: Pop 2, V2.0—2022.02, Revopoint3D, Hong Kong, China) and a second 3D facial scan (RAYFace^®^ 3D scanner, Model: RFS200, Ray Co. Ltd., Hwaseong-si, Republic of Korea). A single measurement for each linear distance was performed. The direct method was considered the “gold standard” as in previous studies [21,33,34].

### 2.3. Measurement Conditions

The ambient light conditions were standardized during the data collection procedure with a set-up with controlled illumination (4100 K). The participants were seated on a chair and were instructed to maintain the same posture during the whole process: natural head position, keeping the eyes open and looking at the horizon with maximum intercuspation position and lips sealed. The posture of each participant was strictly controlled by the operator.

### 2.4. Manual Measurement

**Scheme 2:**
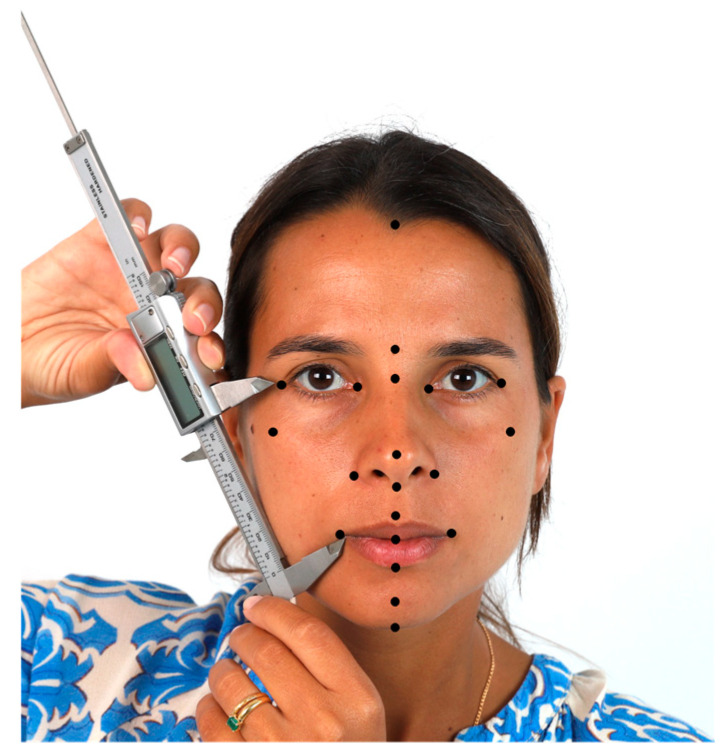
Conventional measurement of a distance with a digital caliper.

For the manual method, anthropometric soft tissue distances were measured with a digital caliper (Perel, 150 mm/6 in, 0.01 mm accuracy) from the center of the market points (Scheme 2) according to previously described methodologies [21,34] in three different planes—the frontal, sagittal and horizontal plane—explained in Table 2.

### 2.5. Digital Acquisition and Measurement

The calibration of the devices was carried out prior to each measurement according to the manufacturer’s instructions to standardize scanner conditions as much as possible.

**Scheme 3:**
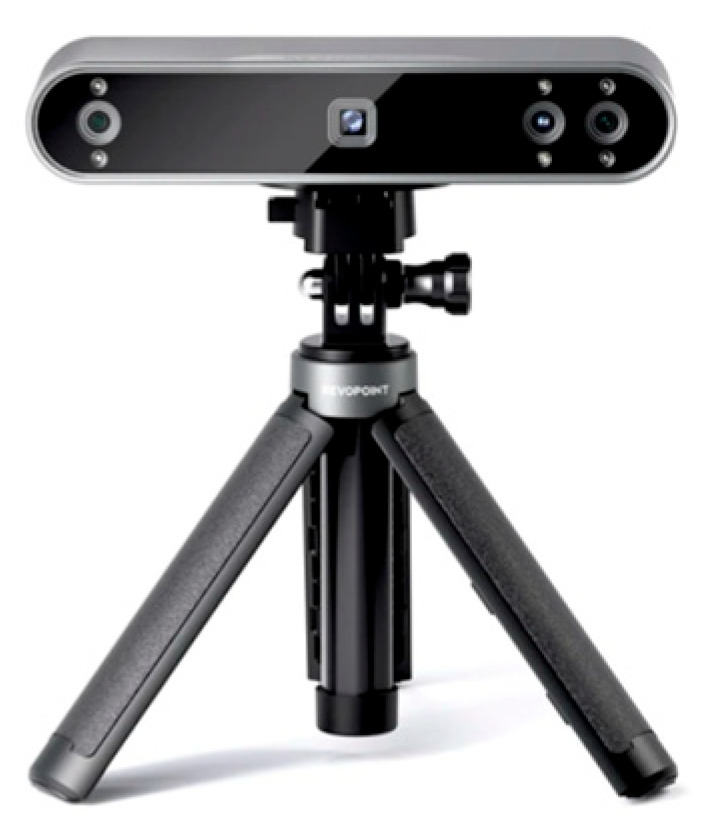
Revopoint^®^ POP 3D scanner.

A standardized set-up was created in a room with controlled illumination (4100 K). Revopoint^®^: The facial scanner (Scheme 3) was connected to a smartphone (Xiaomi, Mi Note 10, 13.0.2.0, Android version 11, Beijing, China). In the main menu a new project was created for each patient.

To keep the optimal distance range for digitalization, the software shows in blue an underexposure and in red an overexposure situation. This distance was controlled by the operator at any moment by the indications of the software.

As previously mentioned, the volunteer was instructed to remain seated, in a chair with support for the dorsal area and the head and neck to remain as still as possible during the acquisition with the portable scanner, looking at the horizon, without following the position of the scanner, which will be moving around the volunteer.

The method of scanning started from one side, moving to the front of the patient and to the other side, including the neck zone, in progressive movements capturing the whole image, with special care in shadow areas, ensuring that they are read in integration.

Considering the final rendering of the image, the textured option was selected.

**Scheme 4:**
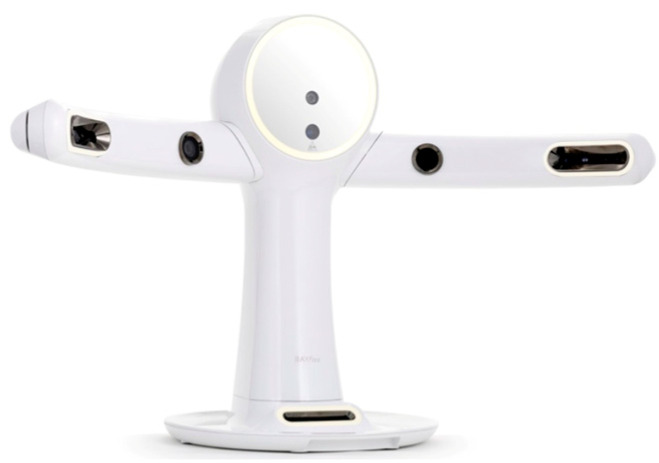
RAYFace^®^ facial scanner.

RAYFace^®^: The facial scanner (Scheme 4) was connected to a computer (Desktop-Q7HL1L, Intel^®^Core™ i7-7700HQ CPU @ 2.80 GHz 2.81 GHz; 16 GB RAM, OS 64 bits; Windows 10 Pro v. 22H2). In the main menu a new project was created for each patient. The cameras were turned on and the volunteer was asked to keep an ideal position according to the tridimensional position of the scanner. The entire face and neck needed to be completely visible on the monitor, as well as the left and right sides of the face including the tragus area. The perioral region was framed in the left and right lateral perspectives with the references of the lip contours present in the software with the aim of improving the patient’s positioning. Once the correct positioning of the volunteer was guaranteed, the scan was performed with just one click, the facial image obtained, and the three-dimensional reconstruction option selected.

**Scheme 5:**
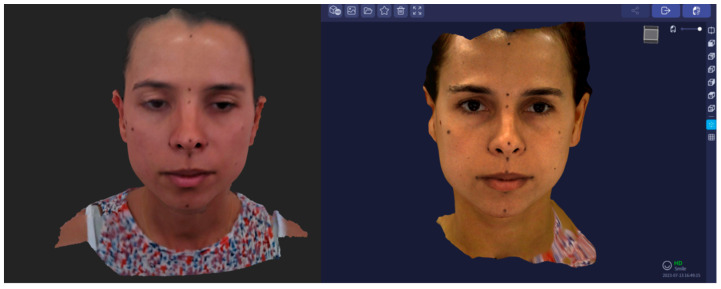
Final tridimensional facial images obtained from Revopoint^®^ and RAYFace^®^, respectively.

The Obj. files obtained from the three-dimensional facial scans of each participant (Scheme 5) were imported into Reverse Engineering software (Geomagic Control X 2022.0.1, 3D Systems, Rock Hill, SC, USA) and calibrated according to the previously described methodologies for digital analysis [25,34]. Briefly, virtual points were marked in the center part of the black landmarks and linear measurements were repeated three times for each location in each patient.

## 3. Statistical Analysis

The outcomes were defined as the trueness and precision of conventional (gold standard) and digital distances in predetermined facial landmarks.

In this study, trueness and precision were defined by previously established methods [21]. Trueness was defined as the mean absolute difference between the manual/gold standard (GS) and digital landmark distances (D_1_, D_2_) calculated as |GS − (average of D_1_; D_2_)|. Precision was defined as the standard deviation (σ) of the different digital measurements (D_1_; D_2_) from each scanner for the same distance, calculated as [σ (D_1_; D_2_)].

Additionally, the trueness impact value in the distance between points was calculated by the formula [|GS − (average of D_1_; D_2_)| × 100]/GS. This represents the size of the difference in the trueness; when we obtained 10% it corresponded to 1 mm of deviation in 10 mm between two points, which means the higher the percentage the greater the impact.

The percentage of precision regarding the landmark distance was calculated by the formula [[σ (D_1_; D_2_)] × 100]/GS.

The sample size was determined a priori for the primary outcome (distance, mm) under a within-subject (paired) design. The expected within-subject variability was derived from a previous in vivo facial scanning accuracy study reporting a global precision of 322.31 ± 300.54 μm (SD ≈ 0.3005 mm) [34]. Assuming a two-sided α = 0.05, 80% power, and a minimum relevant/detectable mean difference of 0.20 mm between methods, a minimum of 20 participants were required. To increase robustness, accommodate potential exclusions, and remain conservative in the context of multiple landmark comparisons, 30 participants were recruited.

The results were presented as means and confidence intervals at 95%, and mean and standard deviation. The normality of distribution was tested by the Shapiro–Wilk normality test, and the Levene test was used to assess the equality of variance. Nonparametric paired Wilcoxon signed-rank test and Friedman test were applied as appropriate. The significance level was established at 0.05. All calculations were carried out with SPSS statistical software (version 29.0.1.1, SPSS, IBM).

## 4. Results

Thirty volunteer participants (29 females and 1 male) aged from 20 to 45 years (mean 30 years) were included in the study. Mean interlandmark distances for manual and digital measurements are presented in Table 3. Both scanners demonstrated reproducible measurements across all evaluated distances. The results obtained from both scanners for each location are explained in Table 4 and Table 5.

Revopoint^®^ exhibited an overall precision of 0.58 [0.53; 0.62] mm, equating to 1.19 (1.06; 1.33) %. The trueness was 2.16 (2.01; 2.31) mm, corresponding to 4.30 [3.87; 4.74] %.

**Figure 1:**
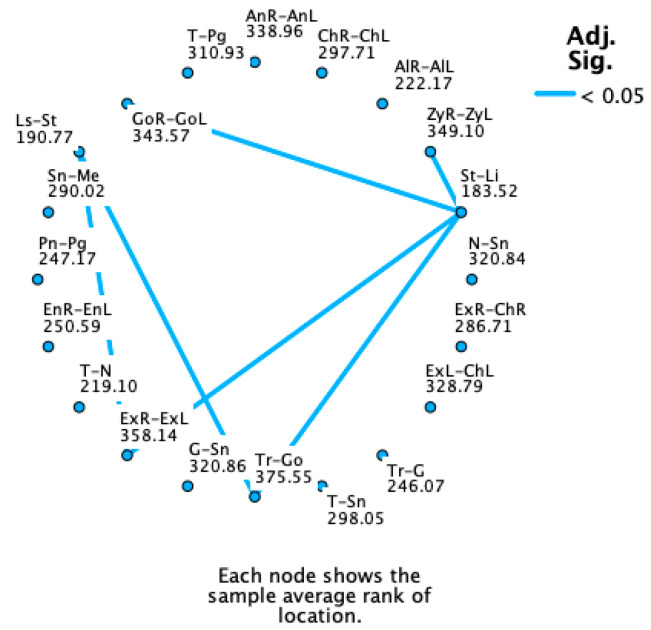
Pairwise comparison method of location (trueness|Revopoint^®^).

**Figure 2:**
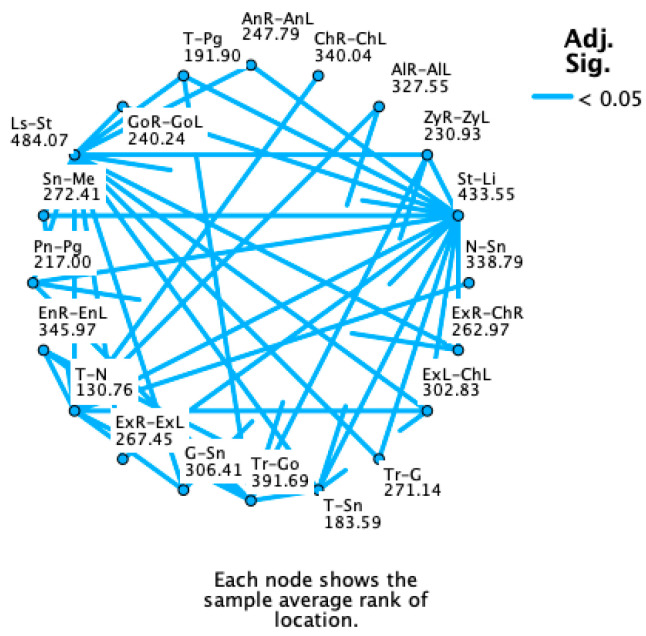
Pairwise comparison method of location (% of trueness|Revopoint^®^).

In the Revopoint^®^ group we observed five locations with statistically significant differences in the trueness (Figure 1) and 36 locations with statistically significant differences in the trueness impact value between them (Figure 2).

**Figure 3:**
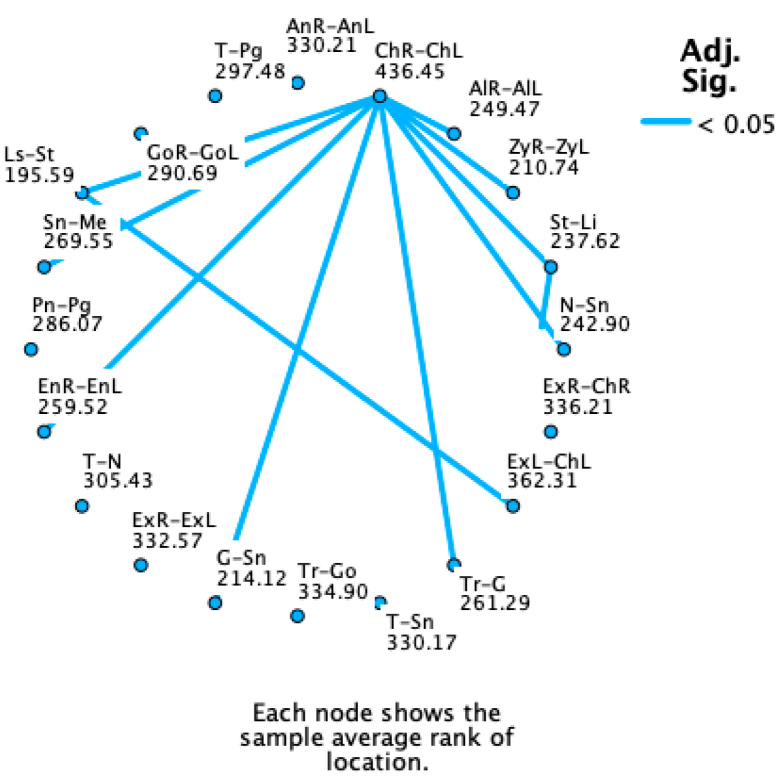
Pairwise comparison method of location (precision|Revopoint^®^).

**Figure 4:**
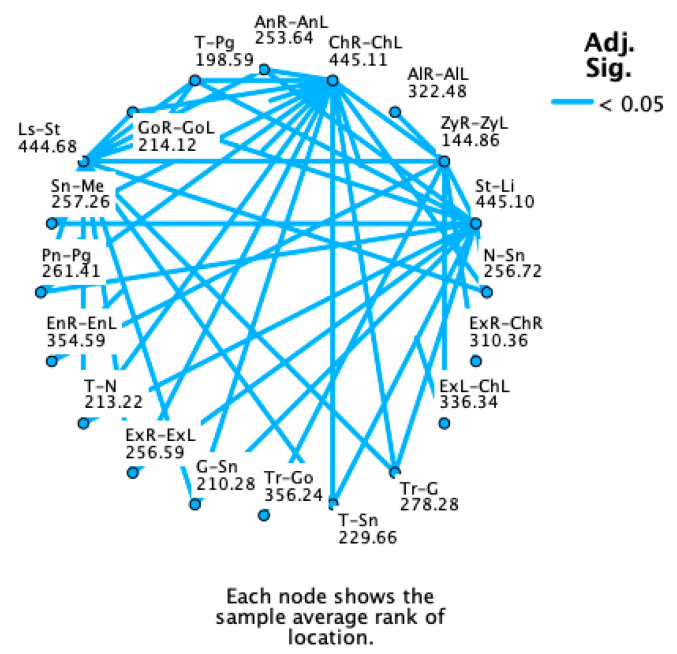
Pairwise comparison method of location (% of precision|Revopoint^®^).

Considering precision, in the Revopoint^®^ group we observed 10 locations with statistically significant differences (Figure 3) and 41 locations with statistically significant differences in the percentage of precision between them (Figure 4).

In the RAYFace^®^ group, the overall precision was 0.43 [0.39; 0.47] mm, equating to a percentage of 0.88 [0.78; 0.97] %.

The trueness was 1.92 [1.80; 2.05] mm, corresponding to 4.08 [3.61; 4.55] %.

**Figure 5:**
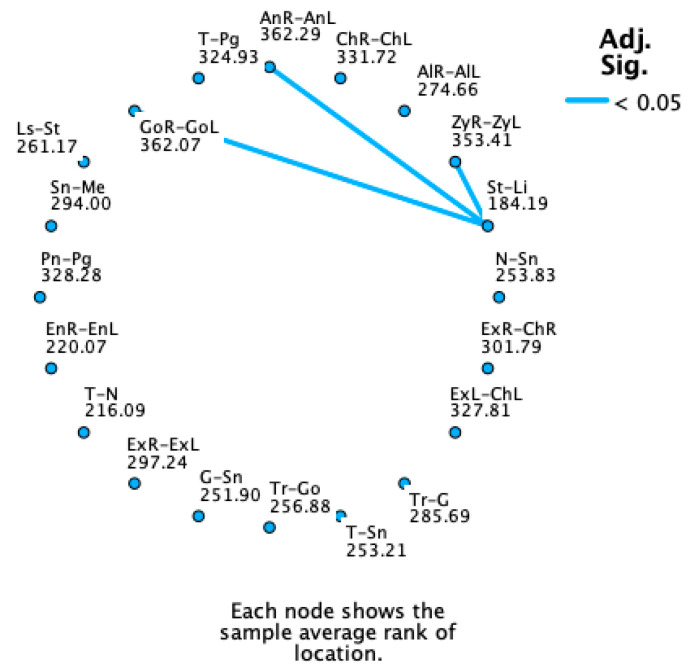
Pairwise comparison method of location (trueness|RAYFace^®^).

**Figure 6:**
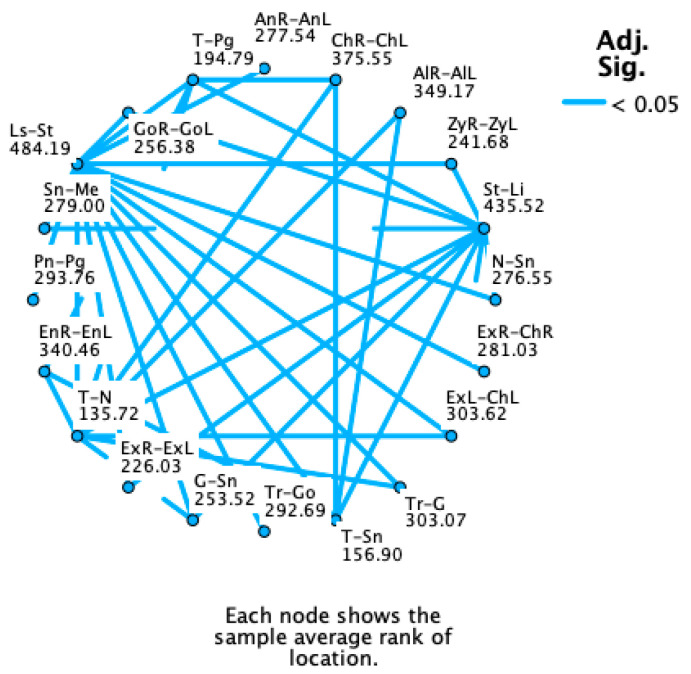
Pairwise comparison method of location (% of trueness|RAYFace^®^).

In the RAYFace^®^ group we observed three locations with statistically significant differences in the trueness (Figure 5) and 32 locations with statistically significant differences in the trueness impact value (Figure 6).

**Figure 7:**
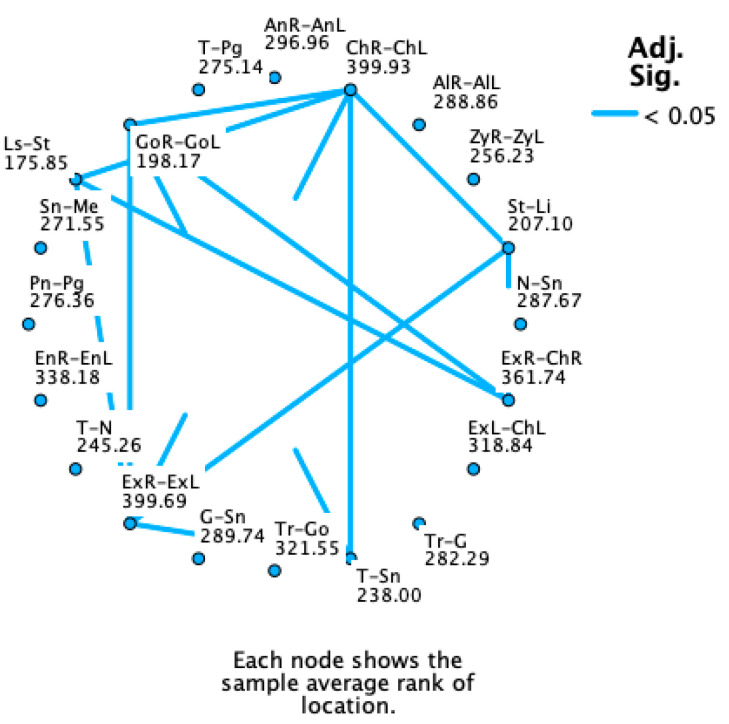
Pairwise comparison method of location (precision|RAYFace^®^).

**Figure 8:**
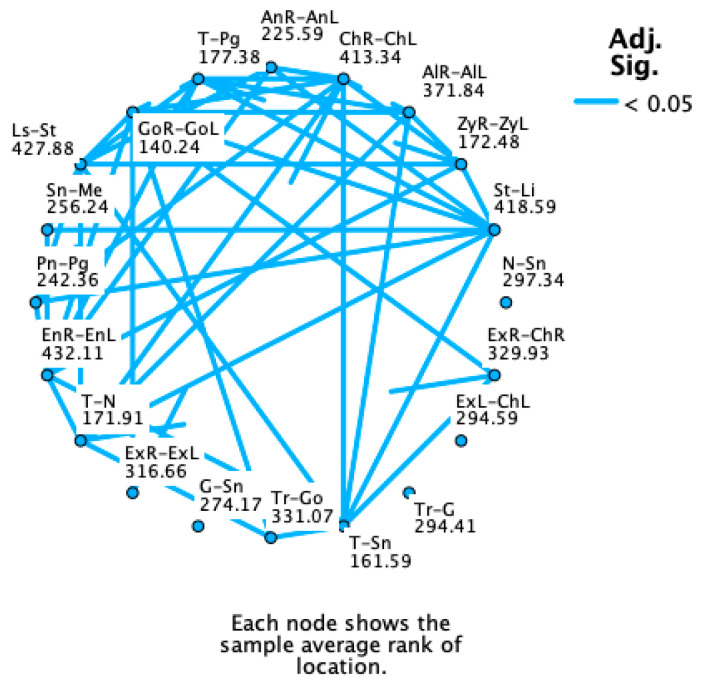
Pairwise comparison method of location (% of precision|RAYFace^®^).

Considering precision, in the RAYFace^®^ group we observed 10 locations with statistically significant differences (Figure 7) and 42 locations with statistically significant differences in the percentage of precision (Figure 8).

The Revopoint^®^ scanner exhibited a mean trueness of approximately 2.16 mm, whereas the RAYFace^®^ demonstrated a statistically lower trueness error (mean trueness of 1.92 mm; paired Wilcoxon signed-rank test, two-sided *p* = 0.013). Both valued remained within the clinically acceptable range up to 2–3 mm, which is particularly relevant for prosthodontic and esthetic planning [9,34].

Measurement errors were positively correlated with interlandmark distance. Landmarks with greater separation, such as gonial and tragion points, showed increased error margins, corroborating previous findings that scanning accuracy decreases with longer spans [35]. In contrast, midline and perioral structures demonstrated higher reproducibility. These regions are critical in smile design and anterior dental rehabilitation, making their accurate capture especially relevant.

## 5. Discussion

The present in vivo study evaluated the trueness and precision of two different facial scanning systems—a low-cost portable device (Revopoint^®^) and a professional static scanner (RAYFace^®^)—using conventional anthropometric measurements as a clinical reference standard for facial linear distance assessment. The results demonstrated that both digital systems provided reproducible linear measurements with clinically acceptable accuracy. Nevertheless, the professional static scanner consistently exhibited higher trueness and precision.

Although manual anthropometry has historically been regarded as a reference method for facial measurements, its use as a benchmark presents inherent limitations, particularly when applied to soft tissues. Its operator dependency, landmark ambiguity, time-consuming nature, soft tissue compressibility and impracticability of recollecting the data once measurements are completed may introduce systematic and random errors that cannot be entirely eliminated [7,8]. In the present study, manual measurements were therefore considered a clinical reference standard rather than an absolute gold standard. This distinction is critical for the appropriate interpretation of the reported trueness values.

The single-operator design was adopted to minimize inter-operator variability and to isolate scanner-related performance. The operator was highly experienced in these types of measurements. Nevertheless, the absence of intra- and inter-observer reliability analyses represents a methodological limitation, as measurement repeatability and potential systematic bias could not be quantified. Future investigations should incorporate reliability testing, such as intraclass correlation coefficients, to further strengthen methodological robustness.

Facial scanning is a technique-sensitive procedure influenced by multiple factors. Patient-related variables such as involuntary head movement, nasal breathing, eye blinking, subtle lip motion and facial muscular contraction may contribute to the distortion of the acquired data. These factors are particularly relevant for portable scanners, which typically require longer acquisition times and continuous movement of the device around the patient, thereby increasing susceptibility to motion artifacts.

Consistent with previous investigations, the present results showed that measurement errors increased with greater interlandmark distances [27,36]. Larger facial spans, particularly those involving gonial and tragion landmarks, demonstrated higher discrepancies, supporting the notion that scanning accuracy decreases as the distance between landmarks increases. In this context, static scanners may offer advantages over portable systems due to their shorter capture time and simultaneous image acquisition, which reduce cumulative errors and motion-related distortion [36].

The transition toward three-dimensional (3D) facial acquisition has been driven by the need to reduce subjectivity and to allow the incorporation of facial data into digital workflows for prosthodontic rehabilitation and interdisciplinary treatment planning [4,5]. Our results are consistent with those of earlier reports indicating that structured light and stereophotogrammetry-based systems can achieve submillimetric accuracy, whereas handheld devices tend to present higher variability related to scanning technique, operator experience and environmental conditions [21,23,28].

In this study, the Revopoint^®^ scanner presented a mean trueness of approximately 2.1 mm, compared to 1.9 mm with the RAYFace^®^. Although statistically significant, these differences remain within the clinically acceptable threshold of 2–3 mm, particularly relevant for prosthodontic and esthetic workflows [9,32,34]. However, these values should not be interpreted uniformly across all clinical applications. While such deviations may be clinically acceptable for smile design, esthetic planning with interdisciplinary communication and prosthodontic rehabilitation, they may be insufficient for applications requiring submillimetric accuracy. This distinction is essential to avoid overgeneralization of clinical suitability.

The anatomical region played a relevant role in scanner performance. Greater distances between landmarks such as those involving gonial or tragion points registered larger errors, supporting prior findings that scanning accuracy decreases over longer facial spans [36]. In contrast, midline or perioral landmarks demonstrated higher reproducibility, which is clinically advantageous given their importance in smile design and rehabilitation.

Patient-related factors also influence scanner performance. Movements like blinking, breathing, involuntary head movement or subtle facial muscle activity can affect digital acquisitions, particularly with portable systems requiring longer scanning times [36]. The static RAYFace^®^ scanner, with its shorter capture time and simultaneous image acquisition, likely mitigates cumulative motion-related errors, which may partially explain its superior performance.

The female predominance of the sample constitutes another important limitation. Facial morphology differs between sexes, and the present findings should therefore be interpreted as primarily applicable to adult female populations. Extrapolation to male patients, older individuals or surgically altered faces should be approached with caution.

The clinical implications of these results are substantial. With the development of facial scanning technology, the integration of facial scans with intraoral and skeletal imaging modalities enables the construction of a “virtual patient”, enhancing diagnostic capabilities and facilitating communication between clinicians, technicians, and patients [6,25]. It allows the collection and analysis of pretreatment and ongoing clinical data [35]. This is particularly relevant in complex prosthodontic treatments, where esthetic and functional outcomes are closely related to the accuracy of facial reference points.

Although it comes with promising results, there are some limitations in this study to be acknowledged. The sample size, though similar to other comparable in vivo studies, remained small, potentially allowing for the presence of selection bias. Additionally, only linear distances were evaluated. Future research should include volumetric and angular analyses, assess longitudinal reproducibility and further investigate the influence of environmental conditions such as lighting variations.

Within these limitations, the present study supports the use of both low-cost and professional face scanners for clinical prosthodontic applications. However, the higher trueness and precision values associated with the RAYFace^®^ suggest that professional systems remain preferable when maximal accuracy is required, particularly in highly complex and high-esthetic-demand cases.

## 6. Conclusions

Within the limitations of this in vivo study, both facial scanners, Revopoint^®^ and RAYFace^®^, provided reproducible facial measurements within clinically acceptable thresholds for prosthodontic and esthetic-planning applications. However, the RAYFace^®^ scanner demonstrated higher trueness and precision and may therefore be more suitable for complex cases such as full-mouth rehabilitation and high-demand esthetic treatments.

However, the findings should be interpreted with caution due to the use of manual anthropometry as a clinical refence standard, the absence of reliability testing and the strong sex imbalance of the sample. The present study represents an incremental, practice-oriented contribution and supports the selective integration of facial scanning technologies into digital prosthodontic workflows. Further in vivo studies are warranted to strengthen the scientific evidence on facial scanning technologies and to support the continued integration of three-dimensional facial data into digital prosthodontic workflows.

## Figures and Tables

**Table 1:** Description of the points marked on the patient’s face regarding the abbreviation used and their location plan.

Point	Abbreviation	Location	Description
Trichion	Tr	Medial	Intersection between the hairline and the midline of the face
Glabella	G	Medial	Most prominent skin point between the eyebrows
Nasion	N	Medial	Point anterior to the nasofrontal suture
Endocanthion	En	Bilateral	Inner corner of the eye
Exocanthion	Ex	Bilateral	Outer corner of the eye
Zygion	Zy	Bilateral	Most lateral point of the zygomatic region
Alare	Al	Bilateral	Most lateral point of the external part of the nose
Pronasale	Pn	Medial	Most prominent point on the tip of the nose
Subnasale	Sn	Medial	Intersection between the nasal septum and the cutaneous portion of the upper lip
Tragion	T	Bilateral	Notch immediately above the tragus
Labrale superius	Ls	Medial	Point at the mucocutaneous junction of the upper lip in the midsagittal plane
Cheilion	Ch	Bilateral	Lip commissure
Stomion	St	Medial	Point of intersection between upper lip and lower lip in the median sagittal plane
Labrale inferius	Li	Medial	Point at the mucocutaneous junction of the lower lip in the midsagittal plane
Pogonion	Pg	Medial	Most anterior point of the chin contour in the median sagittal plane
Gonion	Go	Medial	Most anterior and inferior point of the cutaneous chin
Antegonion	Ag	Bilateral	Most superior point of the concavity of the inferior border of the mandibular ramus
Menton	Me	Medial	Lowest point of the mandibular symphysis

**Table 2:** Description of the distances measured regarding the abbreviation used and its location plan.

Plane	Points	Distance	Definition
Frontal	Trichion Glabella	Tr-G	Height of the upper third of the face
	Glabella Subnasal	G-Sn	Height of the middle third of the face
	Nasion Subnasal	N-Sn	Height of the nose
	Subnasal Menton	Sn-Me	Height of the lower third of the face
	Labrales superius Stomion	Ls-St	Height of the upper lip
	Stomion Labrales inferius	St-Li	Height of the lower lip
	Pronasale Pogonion	Pn-Pg	Distance between the most prominent point of the nose and the most anterior point of the cutaneous chin
Horizontal	Right Exocanthion Left Exocanthion	ExR-ExL	External intercanthal distance
	Right Endocanthion Left Endocanthion	EnR-EnL	Internal intercanthal distance
	Right Zygion Left Zygion	ZyR-ZyL	Facial width
	Right Alare Left Alare	AlR-AlL	Width of the base of the noise
	Right Cheilion Left Cheilion	ChR-ChL	Lip width
	Right Antegonion Left Antegonion	AnR-AnL	Antegonial width
	Right Gonion Left Gonion	GoR-GoL	Lower face width
Sagittal	Tragion Nasion	T-N	Depth of the upper zone
	Tragion Subnasal	T-Sn	Depth of the middle zone
	Tragion Pogonion	T-Pg	Depth of the lower face
	Tragion Gonion	Tr-Go	Length of the mandibular ramus
	Right Exocanthion Right Cheilion	ExR-ChR	Distance between the outermost area of the eye and the lip corner of the corresponding side
	Left Exocanthion Left Cheilion	ExL-ChL	

**Table 3:** Mean values (mm) of the linear distances for all the groups (manual group, Revopoint^®^ group and RAYFace^®^ group); SD, standard deviation.

	Manual Group	Revopoint^®^	RAYFace^®^
Interlandmark Measurement	Mean ± SD	Mean ± SD	Mean ± SD
Tr-G	52.11 ± 7.50	49.99 ± 12.00	50.46 ± 7.12
G-Sn	61.96 ± 4.29	57.87 ± 12.23	60.69 ± 4.44
N-Sn	51.87 ± 4.01	48.09 ± 10.25	50.56 ± 3.94
Sn-Me	64.40 ± 5.09	61.17 ± 12.77	64.21 ± 4.92
Ls-St	7.23 ± 1.26	7.87 ± 2.13	9.01 ± 1.43
St-Li	10.28 ± 2.22	9.28 ± 2.39	9.65 ± 1.51
Pn-Pg	70.38 ± 4.40	67.73 ± 13.81	71.97 ± 4.13
ExR-ExL	92.43 ± 4.35	87.84 ± 17.44	92.42 ± 3.18
EnR-EnL	28.54 ± 2.11	28.89 ± 6.12	28.16 ± 2.06
ZyR-ZyL	109.93 ± 5.03	103.92 ± 20.69	108.57 ± 4.42
AlR-AlL	33.34 ± 2.62	32.12 ± 6.79	35.07 ± 2.56
ChR-ChL	47.02 ± 3.90	44.63 ± 9.89	47.41 ± 3.85
AnR-AnL	98.64 ± 8.67	96.47 ± 20.58	99.25 ± 8.02
GoR-GoL	111.98 ± 7.29	107.33 ± 22.20	111.08 ± 7.82
T-N	113.40 ± 4.19	109.03 ± 21.66	113.05 ± 3.92
T-Sn	115.23 ± 4.29	111.11 ± 22.09	115.39 ± 4.94
T-Pg	126.99 ± 5.09	122.99 ± 24.66	128.47 ± 6.22
Tr-Go	48.78 ± 6.17	46.92 ± 10.51	48.73 ± 5.67
ExR-ChR	66.98 ± 4.82	65.18 ± 13.38	67.48 ± 3.91
ExL-ChL	66.32 ± 3.82	65.84 ± 13.38	67.72 ± 3.79

**Table 4:** Results of trueness and precision (mm; %) obtained from Revopoint^®^ group.

	Conventional-Revopoint^®^			
Interlandmark Measurement	Trueness (mm)	% T	Precision (mm)	% P
Tr-G	1.51 [1.21; 1.82]	2.81 [2.25; 3.38]	0.42 [0.30; 0.53]	0.79 [0.56; 1.02]
G-Sn	2.38 [1.70; 3.07]	3.75 [2.59; 4.91]	0.34 [0.22; 0.46]	0.53 [0.34; 0.72]
N-Sn	2.39 [1.73; 3.06]	4.46 [3.12; 5.81]	0.42 [0.26; 0.59]	0.80 [0.49; 1.11]
Sn-Me	2.09 [1.48; 2.70]	3.11 [2.23; 3.99]	0.50 [0.32; 0.68]	0.78 [0.48; 1.08]
Ls-St	1.15 [0.82; 1.48]	15.64 [11.06; 20.21]	0.28 [0.20; 0.37]	3.81 [2.67; 4.94]
St-Li	1.18 [0.74; 1.62]	10.67 [7.13; 14.21]	0.39 [0.26; 0.52]	3.81 [2.44; 5.18]
Pn-Pg	1.65 [1.17; 2.14]	2.24 [1.62; 2.85]	0.48 [0.36; 0.61]	0.68 [0.49; 0.86]
ExR-ExL	2.72 [2.09; 3.35]	2.83 [2.20; 3.47]	0.60 [0.46; 0.75]	0.63 [0.48; 0.79]
EnR-EnL	1.76 [1.18; 2.33]	6.10 [4.08; 8.12]	0.43 [0.30; 0.55]	1.41 [0.99; 1.83]
ZyR-ZyL	2.69 [2.00; 3.38]	2.35 [1.77; 2.94]	0.34 [0.21; 0.47]	0.30 [0.19; 0.41]
AlR-AlL	1.38 [1.04; 1.72]	4.07 [3.04; 5.09]	0.39 [0.28; 0.50]	1.13 [0.79; 1.47]
ChR-ChL	2.10 [1.53; 2.66]	4.35 [3.19; 5.51]	1.23 [0.88; 1.58]	2.50 [1.82; 3.18]
AnR-AnL	2.89 [1.94; 3.85]	2.89 [1.91; 3.86]	0.72 [0.47; 0.96]	0.72 [0.46; 0.98]
GoR-GoL	3.02 [2.11; 3.93]	2.61 [1.84; 3.37]	0.70 [0.40; 1.00]	0.61 [0.35; 0.88]
T-N	1.45 [0.97; 1.92]	1.24 [0.84; 1.64]	0.63 [0.40; 0.86]	0.54 [0.34; 0.74]
T-Sn	2.23 [1.51; 2.94]	1.87 [1.27; 2.47]	0.73 [0.43; 1.03]	0.62 [0.36; 0.69]
T-Pg	2.59 [1.75; 3.44]	1.96 [1.34; 2.59]	0.68 [0.38; 0.97]	0.52 [0.29; 0.76]
Tr-Go	3.18 [2.40; 3.97]	6.51 [4.72; 8.29]	0.91 [0.51; 1.30]	1.86 [1.01; 2.72]
ExR-ChR	2.13 [1.46; 2.79]	3.05 [2.13; 3.96]	0.65 [0.47; 0.83]	0.94 [0.68; 1.20]
ExL-ChL	2.69 [1.88; 3.50]	3.98 [2.73; 5.23]	0.68 [0.53; 0.83]	0.99 [0.77; 1.21]

**Table 5:** Results of trueness and precision (mm; %) obtained from RAYFace^®^ group.

	Conventional-RAYFace^®^			
Interlandmark Measurement	Trueness (mm)	% T	Precision (mm)	% P
Tr-G	1.93 [1.33; 2.52]	3.49 [2.46; 4.52]	0.50 [0.16; 0.84]	0.89 [0.36; 1.43]
G-Sn	1.52 [1.14; 1.90]	2.38 [1.79; 2.96]	0.39 [0.27; 0.51]	0.60 [0.42; 0.78]
N-Sn	1.62 [1.12; 2.12]	2.98 [2.08; 3.88]	0.37 [0.26; 0.47]	0.70 [0.49; 0.90]
Sn-Me	1.94 [1.40; 2.49]	2.87 [2.08; 3.65]	0.34 [0.24; 0.44]	0.53 [0.36; 0.70]
Ls-St	1.58 [1.16; 2.00]	20.99 [15.52; 26.45]	0.18 [0.12; 0.24]	2.35 [1.60; 3.11]
St-Li	1.07 [0.71; 1.42]	9.64 [6.45; 12.83]	0.23 [0.15; 0.32]	2.20 [1.34; 3.07]
Pn-Pg	2.18 [1.66; 2.70]	2.99 [2.52; 3.71]	0.37 [0.25; 0.50]	0.50 [0.33; 0.68]
ExR-ExL	2.02 [1.42; 2.62]	2.13 [1.48; 2.77]	0.67 [0.52; 0.83]	0.70 [0.54; 0.86]
EnR-EnL	1.32 [0.90; 1.73]	4.42 [3.06; 5.78]	0.49 [0.35; 0.64]	1.68 [1.19; 2.17]
ZyR-ZyL	2.46 [1.88;3.03]	2.17 [1.67; 2.67]	0.30 [0.22; 0.38]	0.27 [0.19; 0.34]
AlR-AlL	1.80 [1.26; 2.34]	5.36 [3.67; 7.05]	0.38 [0.27; 0.50]	1.14 [0.78; 1.51]
ChR-ChL	2.22 [1.66; 2.78]	4.61 [3.39; 5.83]	0.91 [0.62; 1.21]	1.87 [1.26; 2.49]
AnR-AnL	2.81 [2.05; 3.57]	2.85 [2.07; 3.63]	0.45 [0.29; 0.61]	0.45 [0.29; 0.61]
GoR-GoL	2.99 [2.04; 3.96]	2.59 [1.79; 3.39]	0.25 [0.13; 0.38]	0.22 [0.11; 0.33]
T-N	1.36 [0.86; 1.86]	1.15 [0.74; 1.55]	0.44 [0.15; 0. 73]	0.37 [0.13; 0.62]
T-Sn	1.51 [1.14; 1.89]	1.28 [0.96; 1.59]	0.31 [0.19; 0.44]	0.26 [0.16; 0.36]
T-Pg	2.19 [1.61; 2.78]	1.67 [1.23; 2.11]	0.39 [0.24; 0.53]	0.29 [0.18; 0.40]
Tr-Go	1.61 [1.14; 2.08]	3.31 [2.29; 4.32]	0.53 [0.32; 0.73]	1.03 [0.62; 1.44]
ExR-ChR	2.01 [1.46; 2.57]	2.96 [2.13; 3.78]	0.59 [0.43; 0.76]	0.86 [0.62; 1.11]
ExL-ChL	2.29 [1.67; 2.91]	3.41 [2.44; 4.39]	0.49 [0.33; 0.66]	0.72 [0.48; 0.96]

## Data Availability

The data presented in this study are available on request from the corresponding author.
